# A Multi-Channel Borehole Strain Measurement and Acquisition System Based on FPGA

**DOI:** 10.3390/s23156981

**Published:** 2023-08-06

**Authors:** Xin Xu, Zheng Chen, Hong Li, Weiwei Zhan, Wenbo Wang, Yunkai Dong, Liheng Wu, Xiang Li

**Affiliations:** 1 National Institute of Natural Hazards, Beijing 100085, China; xuxin212@mails.ucas.ac.cn (X.X.);; 2 School of Emergency Management Science and Engineering, University of Chinese Academy of Sciences, Beijing 100049, China

**Keywords:** crust deformation observations, multi-channel data acquisition, phase-sensitive detection, borehole strain-meters

## Abstract

In this study, an FPGA(Field Programmable Gate Array)-based borehole strain measurement system was designed that makes extensive use of digital signal processing operations to replace analog circuits. Through the formidable operational capability of FPGA, the sampled data were filtered and denoised to improve the signal-to-noise ratios. Then, with the goal of not reducing observational accuracy, the signal amplification circuit was removed, the excitation voltage was reduced, and the dynamic range of the primary adjustments was expanded to 130 dB. The system’s online compilation function made it more flexible to changes in measurement parameters, allowing it to adapt to various needs. In addition, the efficiency of the equipment use was enhanced. The actual observational results showed that this study’s FPGA-based borehole strain measurement system had a voltage resolution higher than 1 μV. Clear solid tides were successfully recorded in low-frequency bands, and seismic wave strain was accurately recorded in high-frequency bands. The arrival times and seismic phases of the seismic waves S and P were clearly recorded, which met the requirements for geophysical field deformation observations. Therefore, the system proposed in this study is of major significance for future analyses of geophysical and crust deformation observations.

## 1. Introduction

**Figure 1:**
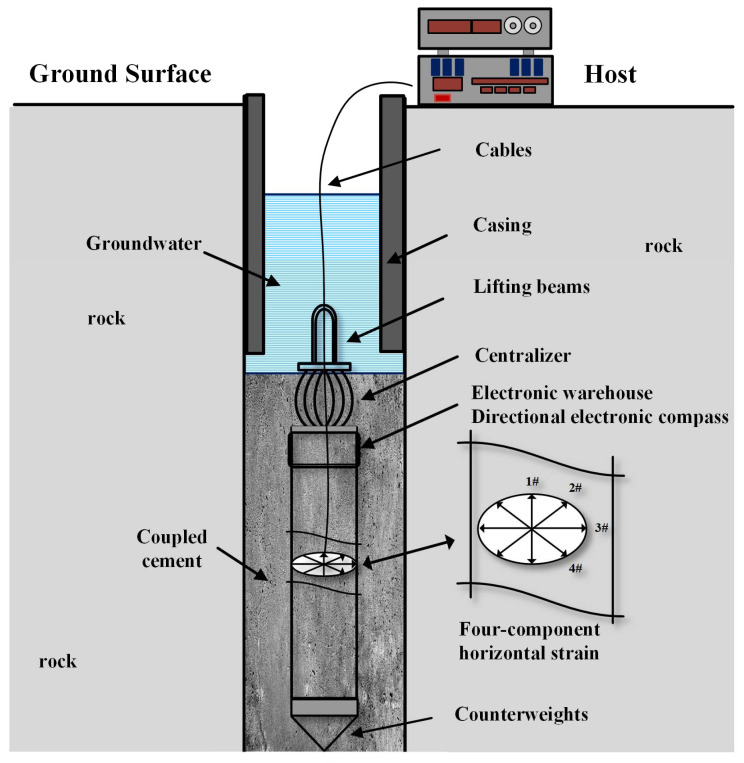
Schematic diagram of a borehole strain measurement system.

Borehole strain observation is a direct mechanical observation method which observes the deformation of the borehole aperture through the displacement sensors installed in the borehole, gives the deformation state of the surrounding rock formation and its dynamic changes, and establishes a mathematical model based on elasticity theory to solve the stress state and its evolution process. Borehole strain-meters are usually composed of three parts: downhole probe, surface host, and data collector [1,2], as shown in Figure 1. The borehole strain-meter has a wide observation band: its high-frequency band (0.1 to several hundred seconds) can be close to the observation band of seismometers, and the low-frequency band (month to year) can cover the observation bands of GPS and InSAR. In its optimal performance of the medium-frequency band (hours~months), the advantage of the borehole strain-meter is that other equipment is difficult to replace, and it is the ideal instrument to reveal short-term (seconds~months) continuous deformation of the earth’s crust, which is an important means of observation of earthquakes and volcanic eruptions before and near to when the phenomena occur [3,4,5,6,7,8,9,10].

In order to meet the needs of geophysical research, the strain resolution of borehole strain-meters should be on the order of 10^−10^ strain (strain refers to the change in displacement divided by the baseline and is dimensionless), with a frequency band covering both direct current and strain seismic wave bands. In the low-frequency bands, solid tides, additional crustal strain, and annual changes can be observed. In the high-frequency bands, strain seismic waves, strain steps, slow earthquakes, etc., can be measured [11].

At present, the sensing unit of the borehole strain-meters generally uses a differential capacitance sensor, in which the three parallel metal pole plates of the sensor constitute two differentially varying capacitors; as the outer barrel of the probe is compressed and stretched, the pole plate spacing changes accordingly, and the capacitance then changes [12]. Together with the ratiometric bridge and the phase-sensitive detector modulation circuit, high sensitivity and broadband measurements are achieved.

**Figure 2:**
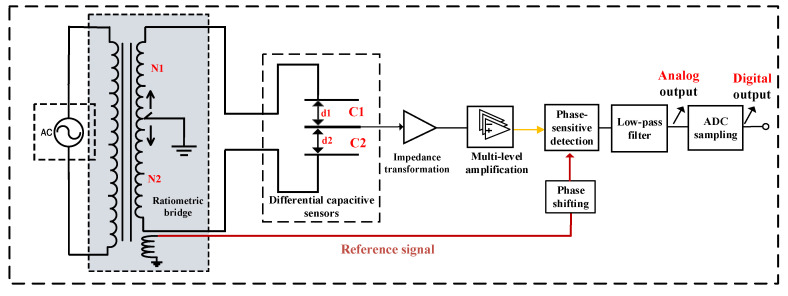
Differential capacitance and ratiometric bridge measurement circuit. In the figure, the peak-to-peak value of the AC excitation is 100 V, with a frequency of 781 Hz. The ratiometric bridge is formed by winding coils, equivalent to 10,000 tap grounding points; AC amplification factor is 100 times. Low-pass filtering cutoff frequency is 30 Hz. ADC sampling frequency is 100 Hz, and the system output resolution is better than 0.1 mV.

As shown in Figure 2, the strain measurement circuits mainly consist of AC excitation, ratio transformers, differential capacitive sensors, impedance transformation, signal amplification, phase-sensitive detection, and low-pass filtering. The ratio transformers and differential capacitive sensors form AC bridges. As the displacements of the capacitor plate change, the unbalanced signal output by the bridge is sent to the AD acquisition unit through impedance transformation, signal amplification, phase-sensitive detection, and low-pass filtering.

Figure 2 shows the classic differential capacitance ratio bridge measurement circuits widely used in small displacement measurements. However, with the popularization of borehole strain observations and the deepening of geophysics research, the shortcomings and limitations of the above-mentioned circuits are gradually emerging.

For example, when the sensor displacements are too large and the unbalance signal of the bridge circuit is limited at the output end, balance adjustments are required in order to adjust the bridge circuit to a balanced state or within a normal working range. Although the total measuring range of current borehole strain-meters reaches 0.5 mm and the dynamic range is above 130 dB, the dynamic range of the primary adjustment is only approximately 90 dB due to the high signal amplification factor. As instruments for geodetic, seismic, and geophysical observations, borehole strain-meters are often installed at seismic stations and are required to make observations on a time scale of several months or years. This may result in significant displacement accumulations in the observed objects. Therefore, when the dynamic range of the primary adjustment is not large enough, the operator is required to perform frequent zero setting operations, resulting in interruptions in the observation process.

The ratiometric bridge is divided into two types of balance adjustment modes: mechanical and electronic. For mechanical adjustments, micron-scale control accuracy adjustment devices are generally applied to pull the capacitor plate back to the balance position. For electronic adjustments, the tap grounding point is adjusted at the ratio transformer end to achieve a balanced state of the bridge circuit, as shown in Figure 2. The mechanical adjustment mode requires high precision and stability of the adjustment mechanism with a precise and complex structure. The tap grounding balance adjustments of a ratio transformer have simple structures, high voltage dividing accuracy, and strong stability. However, a dedicated ratio transformer needs to be wound and a tap grounding adjustment switch needs to be installed, incurring higher hardware costs. For example, taking a ratio transformer equivalent to 10,000 tap grounding points will account for approximately 80% of the total system hardware cost.

In addition, the signal amplification, phase shifting, phase-sensitive detection, low-pass filtering, and so on of the above measurement circuits are all completed using analog devices, which inevitably introduce electro-instrument noise [13,14]. As a result, the measurement accuracy of the circuits will be limited by the performances of the devices. At the same time, the frequency bands, amplification factors, phase shifting, and other parameters of the measurement system are also determined by analog devices and cannot be flexibly adjusted according to the observational needs during use.

However, due to the advantages of high signal processing accuracy and the flexible adjustment of operational parameters, FGPA digital signal processing technology is increasingly replacing analog circuits and becoming a development trend in the electronic technical field.

In this study, an FPGA-based borehole strain measurement system was designed. These types of systems make extensive use of digital signal processing operations to replace analog circuits. Through the formidable operational capability of FPGA, the sampled data can be filtered and denoised to improve the signal-to-noise ratios. Without reducing the accuracy of observations, the signal amplification circuit was removed, the excitation voltage was reduced, and the dynamic range of the primary adjustment was expanded to 130 dB. The tap grounding points of the ratio transformer were reduced to 100 turns, which greatly lowered the hardware expenses. Its online compilation function made the system more flexible to changes in the measurement parameters. Therefore, it could be adapted to various needs, and the efficiency of the equipment use was improved.

## 2. Design of the Borehole Strain Measurement and Acquisition System Based on FPGA

**Figure 3:**
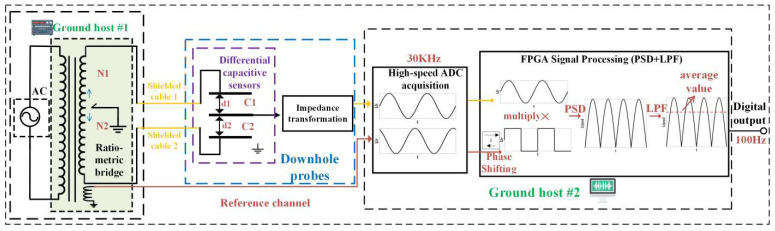
Schematic diagram of the FPGA-based borehole strain measurement system circuits.

This system uses a high-speed, high-precision ADC with FPGA to improve the original borehole strain sensing circuit. As shown in Figure 3, the measuring bridge of the system was composed of a ratio transformer and a differential capacitor. The unbalance signals of the bridge circuit were directly output to the AD acquisition terminal after impedance transformation, and the FPGA controlled the AD to sample the sensing signals and reference signals at high speed (30 KHz). Then, using the powerful operational capability of the FPGA, processing operations such as reconstructions, phase shifting, phase-sensitive detection, digital filtering, and down sampling were carried out. The signals were finally output according to the sampling rate (100 Hz) required for the observations.

### 2.1. High-Precision ADC Control and High-Speed Signal Sampling

In this research investigation, the ADS1256 high-speed and high-precision ADC were used in the proposed system for analog-to-digital conversion. The ADS1256 is a low-noise 24-bit analog-to-digital conversion chip (ADC) with a maximum sampling rate of 30,000 SPS, significant bits of 19.9, noise-free resolution of 17.1 bits, and a main acquisition clock of 50 MHz. Therefore, the ADS1256 met the requirements of sampling and FPGA signal processing. The ADC was connected to the FPGA through an internal SPI serial interface and configured. It read the data by writing registers or commands [15], with a transmission rate of up to 1.5 Mbps.

The main configuration of the ADC was as follows: PGA = 1; analog input buffer closed; differential input; and other settings remained as default. The encoding form for the ADS1256 output is binary complement [16], as detailed in Table 1.

The FPGA selected in this study was the Xilinx A7 series chip XC7A35TFGG484, with a 20 K 6-input lookup table; 40 K D trigger; five independent clock management units; 250 user IO pins; ninety embedded hardware multipliers; and so on. It is rich in resources and can meet the resource requirements of most small- and medium-sized devices.

**Figure 4:**
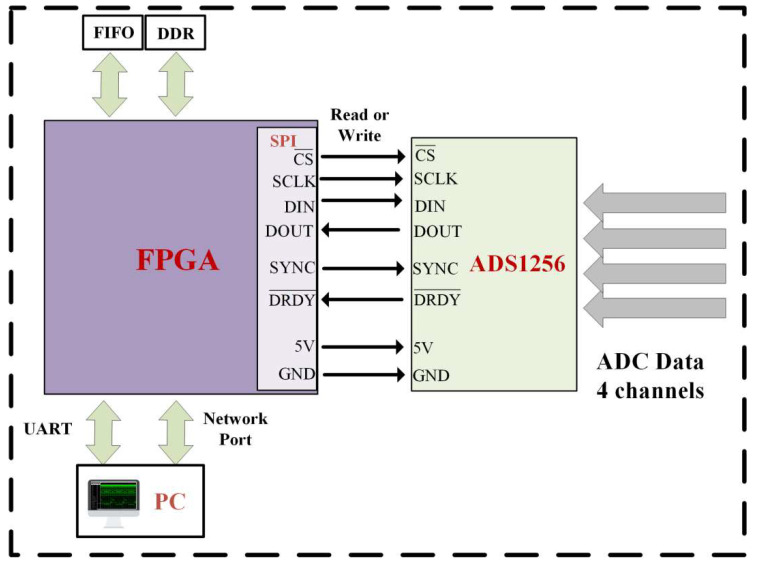
FPGA and ADC control block diagram.

**Figure 5:**
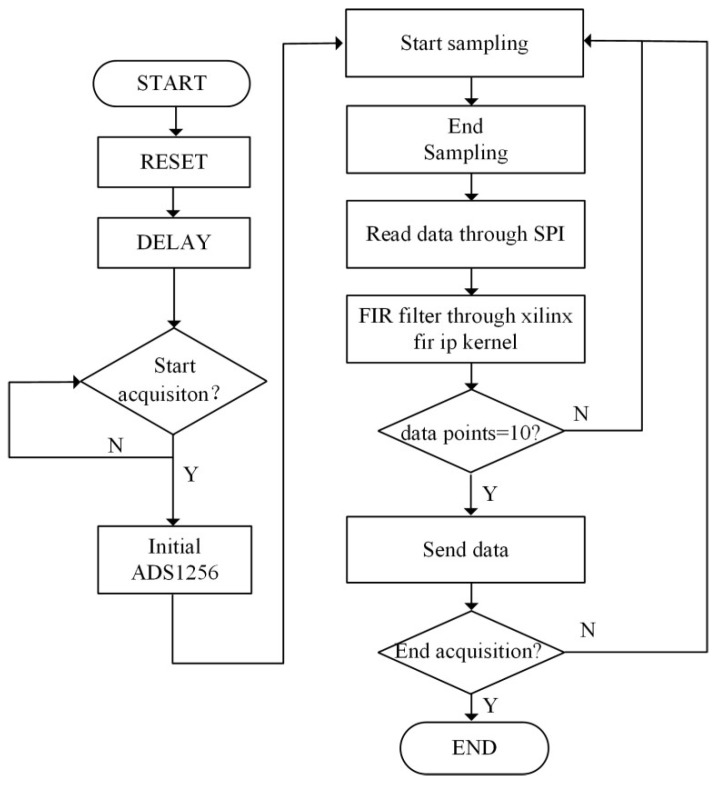
FPGA and ADC acquisition and control flowchart.

The FPGA is controlled by writing code in the Verilog HDL programming language and can achieve functions such as data sampling, processing, transmission, and storage. The system connection and an ADC acquisition flowchart are detailed in Figure 4 and Figure 5.

The specific process of the ADC acquisition module was as follows. After determining that the ADC sampling was enabled to high, the ADS1256 chip was initialized. Then, when the DRDY signal was at a low level, high-speed sampling was performed at a frequency of 30 KHz. Data exchange was carried out with the FPGA through the SPI interface. After completing the phase-sensitive detection, low-pass filtering, and decimation operations in the FPGA, the signal was sent to the upper computer through a network or serial port as needed [17,18]. The baud rate of the serial port was 115,200 bps and the data were 24 bits, which met the data transmission requirements of a 100 Hz sampling frequency.

### 2.2. FPGA Phase-Sensitive Detection

**Figure 6:**
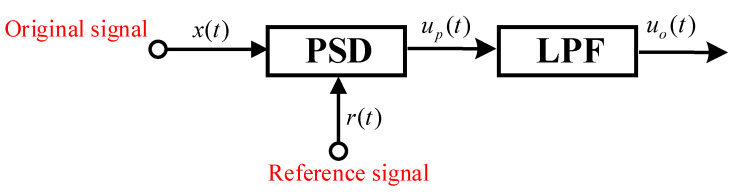
Schematic diagram of the phase-sensitive detection process.

A phase-sensitive detection circuit can be seen as a narrowband filter, mainly consisting of four parts: signal input, reference signal, phase-sensitive detection (PSD), and low-pass filtering (LPF) [19], as shown in Figure 6. The reference signal is a sinusoidal or square wave signal with the same frequency as the signal. A phase-sensitive detector can be regarded as a multiplier which multiplies the reference signal with the carrier signal to obtain the detection signal. However, in order to achieve the best detection effects, the reference signal needs to be phase shifted so that the reference signal is in phase with the modulation signal and the optimal detection results can be obtained [20,21].

When the input signal x(t) is a sinusoidal wave signal and the reference input r(t) is a square wave signal [22], it can be assumed that the input signal form will be as follows:

(1) $$x\left(t\right)=V_{s}cos(\omega_{0}t+\theta )$$

The reference input r(t) is a square wave with an amplitude of $\pm V_{r}$, a period of *T*, and an angular frequency of $\omega_{0}=2\pi /T$. The r(t) can be expressed as a Fourier series as follows:

(2) $$r\left(t\right)=\frac{4V_{r}}{\pi }\sum_{n=1}^{\infty }\frac{{\left(-1\right)}^{n+1}}{2n-1}\cos\left[\left(2n-1\right)\omega_{0}t\right]$$

After phase-sensitive detection and low-pass filtering have been completed, the output will be:

(3) $$u_{0}\left(t\right)=\frac{2V_{s}V_{r}}{\pi }\cos\theta$$

When a single frequency noise with a certain frequency component is doped in the signal, $V_{n}$ is the amplitude of a noise, the following can be assumed:

(4) $$x\left(t\right)=V_{s}\cos\left(\omega_{0}t+\theta \right)+V_{n}\cos\left(\omega_{n}t+\alpha \right)$$

After phase-sensitive detection is completed, the following expression can be obtained:

(5) $$u_{p}=x\left(t\right)r\left(t\right)\;=\frac{2V_{s}V_{r}}{\pi }\sum_{n=1}^{\infty }\frac{{\left(-1\right)}^{n+1}}{2n-1}\cos\left[\left(2n-2\right)\omega_{0}t-\theta \right]+\frac{2V_{s}V_{r}}{\pi }\sum_{n=1}^{\infty }\frac{{\left(-1\right)}^{n+1}}{2n-1}+\cos\left[2n\omega_{0}t+\theta \right]\;+\frac{2V_{n}V_{r}}{\pi }\sum_{n=1}^{\infty }\frac{{\left(-1\right)}^{n+1}}{2n-1}\cos\left[\omega_{n}t+\left(2n-1\right)\omega_{0}t+\alpha \right]\;+\frac{2V_{n}V_{r}}{\pi }\sum_{n=1}^{\infty }\frac{{\left(-1\right)}^{n+1}}{2n-1}\cos\left[\omega_{n}t-\left(2n-1\right)\omega_{0}t+\alpha \right]$$

In Equation (5), the first term has a non-zero output only when n=1, and the fourth term may have a non-zero output. If the frequency of the noise satisfies$\left|\omega_{0}-\omega_{n}\right|<\omega$ (ω is the cutoff frequency of the low-pass filter), the fourth output is $\frac{2V_{n}V_{r}}{\pi }\cos\left[\left(\omega_{n}-\omega_{0}\right)t+\alpha \right]$; noise affects the output and thus reduces the accuracy of the observation. That is, a low-pass filter with a cutoff frequency of w filters out most of the noise, but not the noise that is close to the carrier frequency (the difference is less than ω).

For the FPGA phase-sensitive detection operation in this study, the ADC first samples the unbalance signal of the bridge circuit and the reference signal of the common source excitation. The acquisition test parameters settings are shown in Table 2. Meanwhile, the sampled signal was reconstructed by passing it through a low-pass filter. Its time domain is shown in the following equation:

(6) $$x_{a}\left(t\right)=\sum_{n=-\infty }^{\infty }x\left(n\right)\sin c[f_{s}(t-nT_{s})]$$

where $x_{a}(t)$ is the reconstructed signal and x(n) is the sampled signal sequence. After reconstruction, the number of sampling points in one cycle of the reference signal was interpolated to 1000.

For the processing of the reference signal, it is first filtered with a band-pass filter to remove DC bias and noise; then, it is modulated into a square wave with 50% duty cycle. The center frequency of the bandpass filter was 781 Hz, the same as the excitation, with a passband width of 20 Hz and a stopband width of 200 Hz.

**Figure 7:**
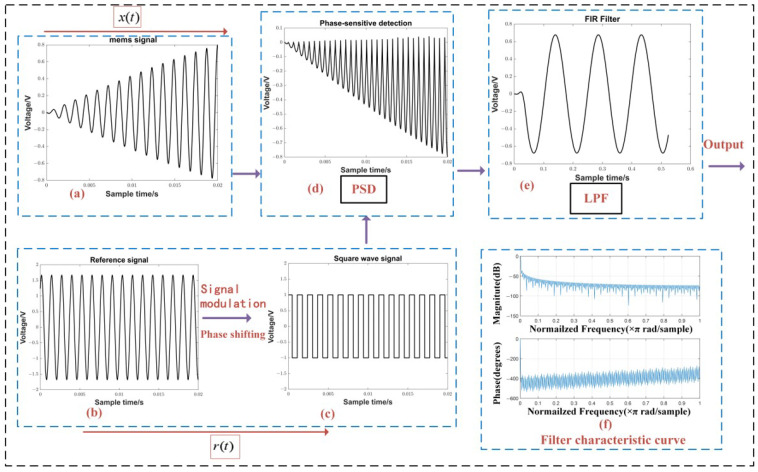
Acquisition test results: (**a**) Sensor signal; (**b**) Reference signal; (**c**) Square wave signal after phase shifting and modulation of the reference signal; (**d**) Detection signal after multiplying the square wave signal and the sensor signal; (**e**) Signal after low-pass filtering; and (**f**) Frequency characteristics of the low-pass filter.

As shown in Figure 6, the reconstructed square wave reference signal was multiplied by the unbalance signal of the bridge circuit to obtain the detection curve. Then, in order to achieve the optimal detection effects of the sinusoidal half-wave, the reference signal needs to be phase shifted through zero padding prior to the phase-sensitive detection. In the simulation shown in Figure 7, it can be seen that the sensor signal had a displacement sensing signal that varied with a pattern of sin⁡(7t). The sensor displacement signal was recovered after phase-sensitive detection and low-pass filtering were completed.

Analog circuits typically achieve phase-sensitive detection using a combination of electronic gating switches, phase shifting, low-pass filtering, and other circuits. The hardware structure is complex. The circuit parameters are determined by analog components, which cannot be flexibly changed. Even worse, the analog device itself tends to be prone to interference (such as phase shifting circuits), resulting in large temperature drifts that easily cause drift noise. However, due to the advantages of high signal processing accuracy and the flexible adjustment of operational parameters, FGPA digital signal processing technology is increasingly replacing analog circuits and becoming a development trend in the electronic technical field.

### 2.3. FPGA FIR Digital Filtering and Down Sampling

Finite impulse response (FIR) filters are commonly used filters for digital signals. With simple structures and linear phases, they are widely used in data acquisition systems [23]. The system function of the M-order FIR filter is as follows:

(7) $$H\left(z\right)=\sum_{k=0}^{M-1}h(k)z^{-k}$$

It is represented by a difference equation, where x(n) is the input sequence and y(n) is the output sequence:

(8) $$y\left(n\right)=\sum_{k=0}^{M-1}h\left(k\right)x\left(n-k\right)=h\left(0\right)x\left(n\right)+h\left(1\right)x\left(n-1\right)+\cdots +h\left(N-1\right)x\left(n-\left(N-1\right)\right)$$

**Figure 8:**
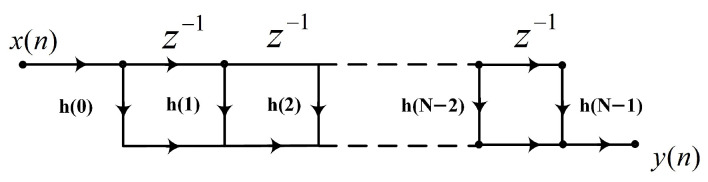
Structure diagram of the direct FIR filter.

The FIR filter is mainly composed of a delay unit, multiplier, and adder [24], where h(m) is the filter coefficient as shown in Figure 8. FIR filtering can be divided into direct, cascaded, distributed, fast convolution, and so on [25].

The system presented in this paper uses the Kaiser window function to design FIR low-pass filters [26]. The observation frequency of borehole strain is in the quasi-direct current in the strain seismic wave frequency range, or between 0 and 30 Hz. Therefore, the order of the FIR low-pass filter was set to 1762, with a passband cutoff frequency of 20 Hz and a stopband cutoff frequency of 40 Hz. Then, after quantifying the generated filter coefficients, a COE file was generated to run on FPGA using Xilinx Fir ip kernel, where the COE file refers to the header file containing the filter coefficients.

After FIR low-pass filtering was completed, the signal carrier and noise were filtered out, leaving only the frequency components less than 30 Hz. The filtered signals were down sampled to 100 SPS by decimation. In this study, due to the existence of the front-end FIR low-pass filter, the signals after down sampling met the Nyquist sampling theorem without aliasing.

## 3. Field Observation Experiments

The FPGA-based borehole strain measurement system was optimized and modified in this study based on the RZB series borehole strain-meters. RZB series of drilling strain gauges have a strain resolution better than 10^−10^ and linearity better than ±1%. The system is compatible with the strain probes of various RZB series borehole strain gauges and can be directly connected to the original strain probes for use.

### 3.1. Selection of Field Observation Site

Gongxian Station was selected as this study’s observation site, which is located in Xunchang Town, Gongxian County, Yibin City, Sichuan Province, and is situated in the southern portion of the Sichuan Basin. In early 2021, an RZB-2 component-type borehole strain-meter was installed to monitor the four horizontal fractional, diagonal, and vertical strains. The bedrock of this installation borehole is relatively intact, the solid tides recorded are clear, and the data quality is good. The station is located in an earthquake-prone zone with frequent peripheral fault activities. As a result, strain steps, slow earthquakes, and other structural activities are often recorded, making Gongxian Station a satisfactory experimental observation site.

**Figure 9:**
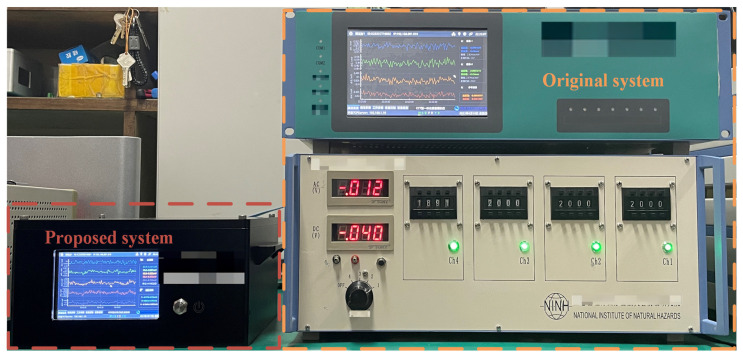
Comparison of the original system and the FPGA-based observation system.

The system on the right side of Figure 9 is a ground observation system composed of the original strain host and the data collector, and the system on the left side of Figure 9 is a borehole strain measurement system based on FPGA. As can be seen in the figure, the FPGA-based borehole strain measurement system greatly reduced hardware expenses, adopted an integrated design, and was compact in size, measuring less than one-third that of the original ground equipment.

### 3.2. System Static Noise Tests

**Figure 10:**
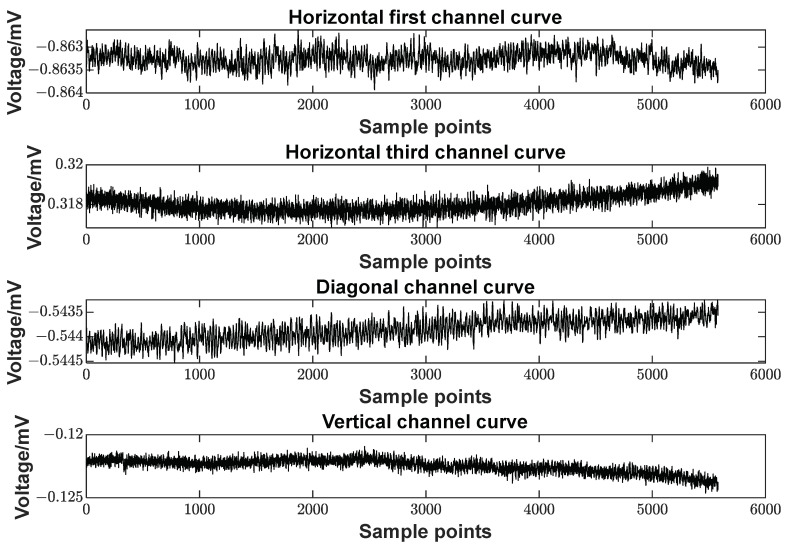
System static noise test results.

The strain measurement resolution of the borehole strain-meters should be on the order of 1~5 × 10^−10^ strain to meet the requirements of geophysical research. In this system, the voltage resolution should be 1.5 μV or higher to fulfill the requirement. Static noise testing was conducted on the data acquisition system. The test results confirmed that the signal-to-noise ratio of the measurement circuit was significantly improved after FIR decimation filtering [27]. The curve is shown in Figure 10. As can be seen in the figure, the static random noise of all four channels of the system was around 1 μV.

In this study, it was found that with the significant improvement of the system resolution, without degrading the observation accuracy, the system no longer needs analog amplification circuitry. The amplitude of the excitation signal could also be lowered to 50 V (PP value). This study’s comparison results of various parameters between the original measurement system and the proposed system are shown in Table 3. Only 100 turns were required by the ratio transformer tap point of the proposed system to achieve a balance adjustment ability of a 250 mV step size.

Borehole strain-meters not only observe the strain seismic wave when an earthquake occurs, but also require a long time to measure the crust of the pressure and tension changes, which is a plate movement observation. The disequilibrium effect of the change in strain on the ratio bridge will slowly build up and ultimately cause a limitation in the output voltage of the measurement circuit. Therefore, the observing equipment needs to have a large dynamic range, while the equilibrium state of each channel needs to be adjusted in some cases to operate near the equilibrium point.

In the original system, the maximum value of the total voltage range at the ADC input is 5 V (−2.5 V–2.5 V), and its measured resolution is 0.1 mv, so the dynamic range is calculated as follows:

(9) $$20\mathrm{lg}\frac{5\;V}{0.1\;\mathrm{mV}}\approx 94\;\mathrm{dB}$$

In this system, the measured resolution is 1 μv, so the dynamic range is calculated as follows:

(10) $$20\mathrm{lg}\frac{5\;V}{1\;\mu V}\approx 134\;\mathrm{dB}$$

The ADC input voltage range may be less than 5 V maximum, so the dynamic range is close to 90 dB or 130 dB.

The on-site programming characteristics of the FPGA allowed for more flexible changes in measurement parameters, as well as being adaptive to various needs and achieving significant improvement in observation efficiency.

### 3.3. Experimental Result

**Figure 11:**
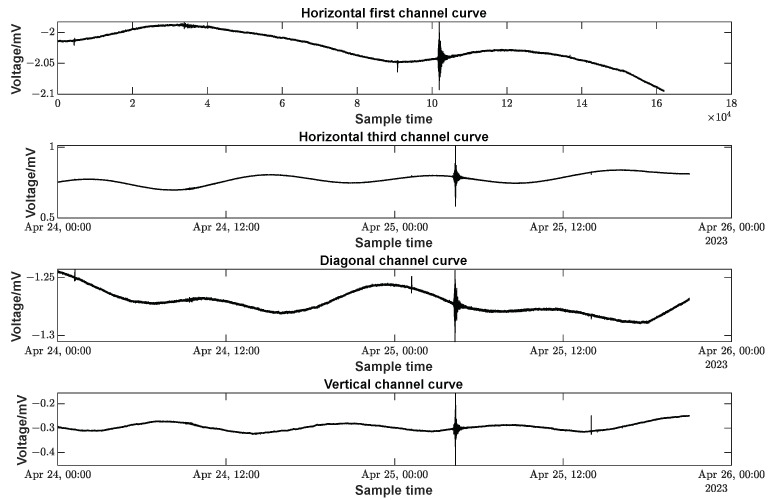
Measured data curve of the acquisition system.

In this study’s actual experimental tests, the system excitation signal frequency was set as 781 Hz; excitation signal amplitude was 50 V (peak-to-peak); and the sampling rate was 100 Hz. Figure 11 shows the voltage data curves of the four strain components, starting from 00:00 on 24 April 2023, for a total of 45 h of sampling. The earthquake recorded in the figure was a 6.9 magnitude earthquake that occurred in the southern waters of Sumatra Island, Indonesia (0.8 degrees south latitude and 98.7 degrees east longitude), with a focal depth of 10 km.

According to the industry standard, in the field of strain observation, it is difficult to obtain an accurate signal source for small displacements. Generally, the theoretical solid tide is used as a benchmark for calibration. Through the measured data and theoretical calculations obtained in comparing the solid tides, the system sensitivity can be obtained. For differential capacitance sensors, laboratory calibration is required before installation [12,28].

### 3.4. Preliminary Analysis of Experimental Results

**Figure 12:**
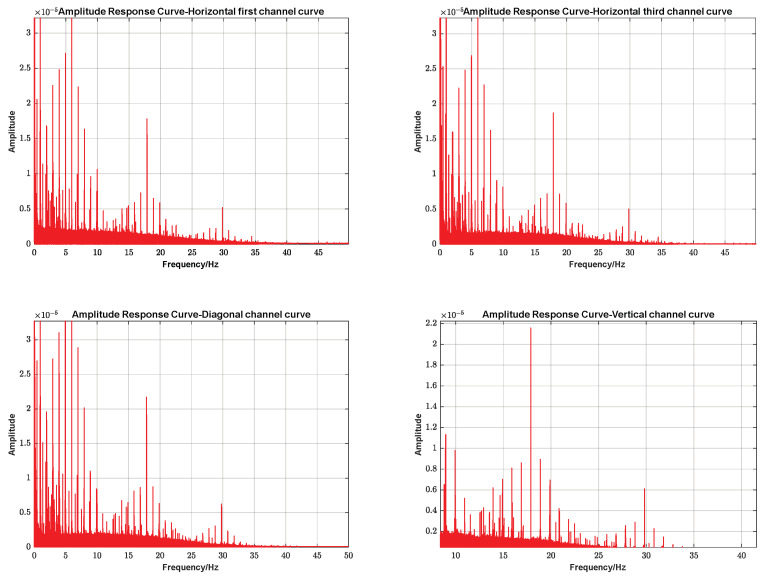
Amplitude response curves for four channels.

Solid tide refers to the phenomenon of periodic deformation of the solid earth under the action of the gravitational tidal force of the sun and the moon: the period of a tidal wave is about 23 h and 48 min, and the frequency is very low, approximating to 0 Hz. A spectral analysis of the data from the four channels mentioned above is shown in Figure 12.

As can be seen from the graphs, the low-frequency portion has a higher weighting, but the influence of many noises and external factors embedded in the data leads to the presence of other frequency components as well.

The observations recorded by the borehole strain-meters can be converted to strain values $S_{i}(i=1,2,3,4)$ in the direction of each element in the inner wall of the instrument by means of laboratory calibration. $S_{i}$ is related to the principal strain of the formation as follows [29,30,31]:

(11) $$S_{i}=A\left(\varepsilon_{1}+\varepsilon_{2}\right)+B\left(\varepsilon_{1}-\varepsilon_{2}\right)\cos2\left(\theta_{i}-\varphi \right)$$

In the above equation, $\varepsilon_{1}$ and $\varepsilon_{2}$ are the maximum and minimum principal strains, respectively; A and B are the coupling coefficients of surface strain and differential strain, respectively; θ is the azimuth angle of the corresponding element of the borehole strain-meters; φ is the azimuth angle of the maximum principal strain; and i is the number of each element of the borehole strain-meters.

**Figure 13:**
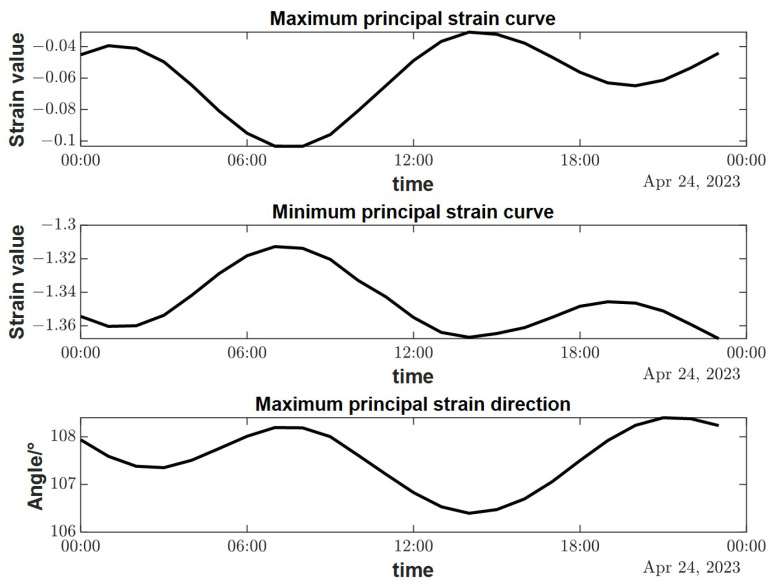
Variation curve of the main strain of the formation calculated from the observation data of the borehole strain-meters.

Figure 13 shows the variation curves of the maximum principal strain $\varepsilon_{1}$, the minimum principal strain $\varepsilon_{2}$; and the maximum principal strain azimuth φ calculated from Equation (9), with a starting moment of 00:00 on 24 April 2023 and a duration of 1 day. From the figure, we can see that the calculated principal strain and its orientation curve have a good signal-to-noise ratio. We can see the obvious solid tides change, which indicates that the FPGA-based borehole strain measurement and acquisition system researched in this paper has a good performance in the low-frequency band; it can be used to calculate the change in magnitude and direction of the principal strain in the formation.

The coupling coefficients A and B respond to the influence of the outer barrel of the borehole strain-meters and the cement ring on the stress–strain near the borehole; they have a very complicated relationship with the elastic modulus and Poisson’s ratio of the outer barrel of the strain-meters, the cement ring, and the surrounding rock; the calibration of these parameters is difficult and requires sampling and testing of the cores near the probe, which has not been performed by most of the observatories in China at present. To simplify the calculation, A and B are taken as 0.5 according to the non-porous rock model in the calculation of Figure 13. Although the results may produce some errors, they do not affect the qualitative analysis of them.

**Figure 14:**
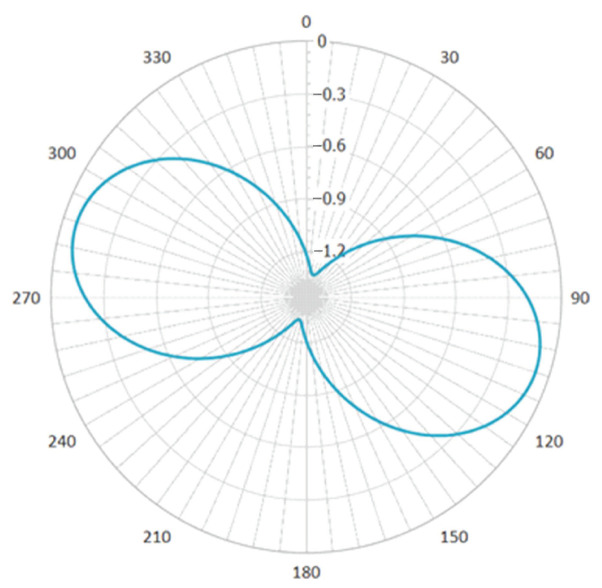
Azimuthal rose diagram of the full range response of a borehole strain-meters.

Figure 14 shows the response of the borehole strain-meters to the main strain of the formation in a full 360-degree range, calculated from the results in Figure 13 and from Equation (11), which can be used for subsequent studies such as anisotropy of the tidal response.

**Figure 15:**
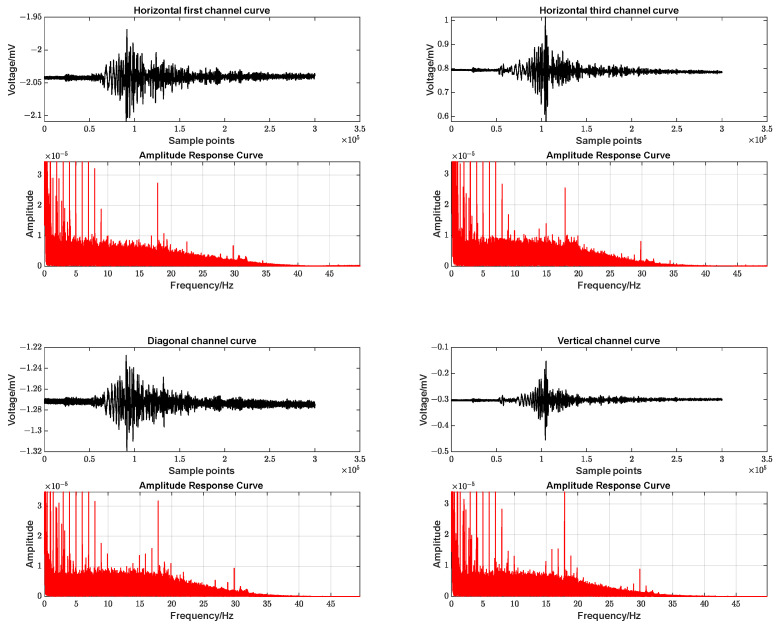
Measured data curves and amplitude response curves before and after a seismic event.

Then, a Fourier analysis was performed for the time period of the earthquake, and the results are shown in Figure 15:

As indicated by the actual observational data in Figure 11 and Figure 15, the FPGA-based borehole strain measurement system achieved a data output sampling rate of 100 SPS. Overall, low frequencies (close to 0 Hz) make up the major portion. If we only look at the time before and after the earthquake, the high frequency will have more weight. Side by side, it verifies that the solid wave is mainly concentrated in the low-frequency component. The seismic strain wave is mainly reflected in the high-frequency component.

**Figure 16:**
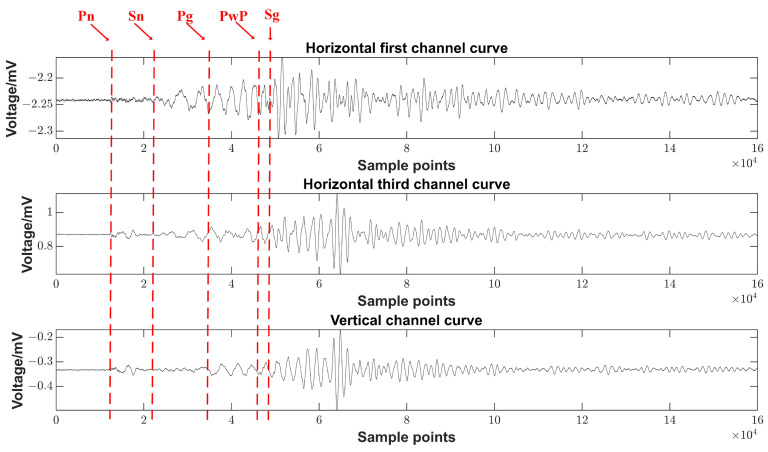
Schematic diagram of the seismic phases recorded by the curve.

Selecting the relevant data and curves of the horizontal first channel, the horizontal third channel, the vertical channel, and the labels of the seismic phase are shown in Figure 16.

Clear solid tides were recorded in the low-frequency bands and strain seismic waves were recorded in the high-frequency bands. The arrival times and seismic phases of the seismic waves S and P were recorded clearly. Therefore, the system met the design requirements.

## 4. Conclusions

In this study, high-speed AD sampling and FPGA digital signal processing techniques were used to improve a borehole strain ratio measurement system. Due to the powerful operational capability of the FPGA, it has not only extensively replaced traditional analog circuits and improved signal resolution but has also eliminated the need for analog signal amplification circuits. The amplitudes of excitation signals could be reduced twice while the resolution quality increased. In this study, without reducing the observational accuracy, the dynamic range of the system increased from 90 dB to 130 dB. Moreover, the tap grounding point of the ratio transformer was reduced from 10,000 turns to 100 turns. After the dynamic range of primary adjustment was expanded, it could be calculated based on the annual variation of the borehole strain observed at the time [32]. In this study, after the equipment was stably installed, a balance adjustment was completed, and there was no need to make any further adjustments within 5 to 8 years. Therefore, the frequency of equipment operational interruptions was reduced. In addition, with the online compilation function of the FPGA, the measurement parameters can be changed online, thereby enhancing the flexibility and adaptability of the equipment and improving the usage efficiency.

In this investigation, according to the actual observational results, the FPGA-based borehole strain measurement system had a voltage resolution around 1 μV. Clear solid tides could be recorded in the low-frequency bands; strain seismic waves could be recorded in the high-frequency bands; arrival times and seismic phases of seismic waves S and P were clearly recorded; and the requirements for geophysical field deformation observations were met [33,34]. Therefore, the proposed system is of major significance for future analyses of geophysical and crust deformation observations.

## Figures and Tables

**Table 1:** ADS1256 output format.

Differential Input/V	Output Binary Encoding
$\geq \frac{5}{\mathrm{PGA}}$	7FFFFFH
$\frac{5}{\mathrm{PGA}(2^{23}-1)}$	000001H
0	000000H
$-\frac{5}{\mathrm{PGA}(2^{23}-1)}$	FFFFFFH
$\leq \frac{5}{\mathrm{PGA}}(\frac{2^{23}}{2^{23}-1})$	800000H

**Table 2:** Acquisition test parameter settings.

Parameters	Symbols	Values
Carrier frequency	*f*	781 Hz
Sampling frequency	*f_s_*	30,000 Hz
Sampling time	*t*	0.52 s
Cutoff frequency of FIR low-pass filter	ω	30 Hz
Sampling time	*T_s_*	1/30,000 s

**Table 3:** Comparison of performance parameters between the original measurement system and the proposed system.

	Proposed System	Original System
Peak-to-peak value of excitation	50 V	100 V
Signal amplification factor	1	100
Required system resolution	1.5 μV	0.15 mV
Measured system resolution	1 μV	0.1 mV
Ratio transformer tap point	100	10,000
Ratio transformer tap grounding point adjustment step size	0.25 V	0.5 V
Maximum zero shift of zero setting	0.125 V	0.25 V
Dynamic range	130 dB	90 dB

## Data Availability

Not applicable.
